# Parental Phubbing and Problematic Mobile Phone Use Among Chinese Early Adolescents: A Longitudinal Moderated Mediation Model

**DOI:** 10.3390/bs16101947

**Published:** 2026-10-20

**Authors:** Shutao Ma, Wei Zhang

**Affiliations:** 1 School of Education Science, South China Normal University, Guangzhou 510631, China; mashutao3319@163.com; 2 Center for Studies of Psychological Application, School of Psychology, South China Normal University, Guangzhou 510631, China

**Keywords:** Chinese early adolescents, parental phubbing, problematic mobile phone use, core self-evaluation, peer relationships

## Abstract

Adolescence is a high-risk period for problematic mobile phone use (PMPU), which exerts severe adverse impacts on adolescents’ physical and mental health. Cross-sectional studies have identified parental phubbing as a core predictor of adolescent PMPU, yet longitudinal evidence for this association remains scarce, as do investigations into protective factors that may attenuate this link. Grounded in Davis’ cognitive-behavioral model and Bronfenbrenner’s ecological systems theory, this one-year three-wave longitudinal study examined the mediating role of core self-evaluation in the relationship between parental phubbing and adolescent PMPU, as well as the moderating role of peer relationships in this mediational process. A sample of 807 early adolescents from southern China (52.4% female; Mage = 12.04 years at baseline) completed self-report assessments at six-month intervals. The results of path analysis, adjusting for age, gender, and family socioeconomic status, showed that parental phubbing at Time 1 significantly predicted higher PMPU at Time 3, and this longitudinal association was significantly mediated by core self-evaluation at Time 2, with peer relationships moderating both the direct path from parental phubbing to PMPU and the first stage of the mediation effect (parental phubbing on core self-evaluation). Specifically, the associations of parental phubbing with core self-evaluation and subsequent PMPU were significantly stronger among adolescents with poor peer relationships, whereas these associations were attenuated for those with high-quality peer relationships. This study reveals a moderated mediation pattern underlying the longitudinal association of parental phubbing with Chinese adolescents’ PMPU, and offers culturally informed considerations for the prevention of adolescent PMPU within the Chinese cultural context.

## 1. Introduction

Adolescents’ increasing preoccupation with their mobile devices has drawn significant attention from a variety of societal sectors. Driven by their strong inquisitiveness and thirst for novelty, teenagers are especially receptive to new technologies, but they also face heightened emotional and behavioral challenges that make them more vulnerable to problematic mobile phone use (PMPU; W. Hong et al., 2019). According to data collected in December 2024, 99.7% of Internet users in China utilize their mobile phones to access the web; among these users, individuals aged 10 to 19 years old make up 13.0% of the total user base (China Internet Network Information Center [CNNIC], 2025). PMPU is characterized by excessive, poorly controlled use of mobile devices and by a dependence that disrupts daily functioning (Lin & Liu, 2020). This pathological pattern differs from ordinary time spent on a mobile phone or simple reliance on mobile devices for everyday activities (F. Y. Hong et al., 2012). PMPU has been associated with anxiety, depression, stress, impulsivity, and poor sleep quality, although the strongest pooled evidence comes from college-student samples (Li et al., 2020, a systematic review and meta-analysis of college students); corresponding evidence in adolescent samples is accumulating (e.g., W. Hong et al., 2019). Identifying the determinants and underlying mechanisms of adolescent PMPU is therefore important for both practice and the development of effective prevention and intervention strategies.

Exposure to and use of digital devices is itself developmentally graded: the correlates of device exposure differ markedly across developmental stages, and findings obtained in one age band cannot be extrapolated to another (Operto et al., 2020). This highlights the value of examining PMPU within a developmentally specific window such as early adolescence.

### 1.1. Parental Phubbing in Relation to PMPU

Previous research has emphasized the important roles of both contextual factors and personal attributes in shaping PMPU (Chiu, 2014). Among these, the family unit, as a fundamental and intimate social structure, plays a crucial role in shaping the development of individuals. Indeed, Q. Q. Liu et al. (2020) have already demonstrated an association between family factors and PMPU. Stage-environment fit theory suggests that a balanced, supportive, and nurturing family environment is crucial for meeting adolescents’ psychological needs, including competence, affiliation, and autonomy, thereby fostering their overall well-being; conversely, an adverse family setting can obstruct the satisfaction of these needs, potentially leading to negative behaviors (Gutman & Eccles, 2007). Parental phubbing refers to parents neglecting face-to-face interactions with their children in favor of attending to their mobile devices (X. Wang et al., 2020). Persistent parental phubbing, which results in children feeling neglected and unable to alter the situation, often drives these children to use their smartphones as a form of escape, thereby increasing their likelihood of PMPU (Xie et al., 2019). Furthermore, the intergenerational transmission effect suggests that parents’ psychological traits and behaviors are transmitted to their offspring, thereby shaping their children’s unique developmental trajectories (Nower et al., 2022). As parents are usually the most consistently present socializing agents in a child’s life, frequent parental phubbing during daily interactions provides children with adverse behavioral models. Prolonged exposure to such a family environment, with ongoing examples of unhealthy parental behaviors, might render adolescents more susceptible to adopting similar negative behaviors, such as PMPU (Dou et al., 2020; Niu et al., 2020). While prior investigations have examined the association between parental phubbing and PMPU, the fundamental processes involved are still not fully understood. Given that adolescence is a crucial developmental phase with long-lasting impacts throughout one’s adult life (Q. Q. Liu et al., 2020; Nower et al., 2022), a cross-sectional study is inadequate for examining how the association between parental phubbing and PMPU unfolds over time. Furthermore, due to imbalances between physical and mental development during this stage of life, adolescents often experience tensions between maturity and immaturity, producing dynamic changes in their psychology and behavior (W. Hong et al., 2019). To address these gaps, the objective of the current study was to explore the longitudinal associations between parental phubbing and PMPU over a one-year period to clarify how the relationship unfolds and to identify potential points for intervention.

### 1.2. The Mediating Role of Core Self-Evaluation

Core self-evaluation refers to individuals’ fundamental appraisals of their own worth and competence, including aspects such as self-esteem, general self-efficacy, neuroticism, and locus of control (Judge et al., 1998). Core self-evaluation is closely associated with key life events and can be shaped or transformed by environmental influences and the assimilation of external information (Judge, 2009). As an aspect of self-concept, core self-evaluation is susceptible to the perceptions of one’s significant others, which are particularly influential during adolescence when self-concept development is still maturing (Koumoundourou et al., 2011). Parents hold a crucial role here, and their negative actions can instill feelings of rejection or neglect in adolescents (Xie & Xie, 2020), damaging their self-worth and diminishing their level of core self-evaluation (X. Wang et al., 2022). Meanwhile, a low level of core self-evaluation increases one’s vulnerability to PMPU (Zhou et al., 2022). Research indicates that individuals exhibiting a reduced level of core self-evaluation tend to employ avoidant coping strategies more frequently (Ding et al., 2022, employee sample), with mobile phone use as one such strategy (Enez Darcin et al., 2016, adult sample); whether these patterns generalize to adolescents is examined here. Studies with college and adult samples have likewise linked the four aspects of core self-evaluation to problematic phone use (Kim & Koh, 2018; Park et al., 2013; Roberts et al., 2015); the present study extends this evidence to Chinese early adolescents. According to Davis’ cognitive-behavioral model, Internet addiction is influenced by distal factors, such as life events, which lead individuals to develop corresponding maladaptive cognitions, namely proximal factors (i.e., core self-evaluation), which subsequently manifest in the behavioral symptom of Internet addiction (Davis, 2001). As forms of behavioral addiction, PMPU and Internet addiction share certain attributes and nosological features (Hadlington, 2015); these theoretical links are drawn cautiously, as PMPU was assessed by self-report questionnaire rather than clinical diagnosis. Thus, this study hypothesized that adolescents’ core self-evaluation mediates the connection between parental phubbing and PMPU, indicating that increased exposure to parental phubbing may decrease adolescents’ level of core self-evaluation, thereby increasing their likelihood of developing PMPU.

### 1.3. Peer Relationships as a Moderator

The association between parental phubbing and adolescent PMPU may be mediated by core self-evaluation, yet not every individual affected by parental phubbing will exhibit low core self-evaluation or PMPU. Ecological systems theory indicates that family and peer influences are interdependent, both acting as micro-systems in shaping adolescents’ development as they interact to exert systemic effects (Bronfenbrenner, 1979). As individuals age, the influence of peer relationships on adolescents in particular becomes progressively more dominant in comparison with that of the family environment (Oberle et al., 2018). Peer relationships refer to interactions among equals or those at similar stages of development (Huang et al., 2022). Previous studies have suggested that strong friendships among peers can greatly reduce the likelihood of adolescents engaging in PMPU (P. Wang et al., 2017), while other studies have found that those experiencing peer aggression or poor relationships are more prone to PMPU (Chen et al., 2021b). The risk-buffering model posits that positive factors in the environment can mitigate the adverse effects of risk factors (Hollister-Wagner et al., 2001). As a positive aspect of one’s living environment, good peer relationships can serve as a protective factor when individuals experience negative events, and buffer the impact of a negative family environment on adolescent problem behavior (Woods et al., 2009). This means that, while parental phubbing may be associated with adverse behavioral outcomes, including increased PMPU, if individuals affected by parental phubbing behavior can establish high-quality peer relationships, they can obtain additional social support, which can help alleviate the stress brought about by these risk factors and reduce the occurrence of PMPU. Conversely, if individuals affected by parental phubbing behavior have low-quality peer relationships, this may exacerbate the harm associated with existing risk factors, making them more prone to developing PMPU (P. Wang et al., 2017). Therefore, this study hypothesized that peer relationships may moderate the association between parental phubbing and PMPU.

Peer relationships are also closely associated with one’s core self-evaluation, as peer relationships can help regulate the impact of familial risk factors on one’s internalizing problems (H. Wang et al., 2023). Adolescents who experience a negative family environment can, however, have additional positive experiences through high-quality peer relationships, which will help increase their self-esteem. If they have low-quality peer relationships, the resulting negative experiences will lead to a lower level of self-esteem (Huang et al., 2022). Sociometer theory posits that self-esteem acts as a social thermometer, indicating the extent to which an individual is accepted and valued by others (Leary & Baumeister, 2000). Adolescents develop higher self-esteem when they experience care and affection, whereas they experience lower self-esteem when they encounter rejection and neglect. Adolescents who have experienced parental phubbing behavior can still receive positive experiences and develop higher self-esteem if they have high-quality peer relationships; however, if they have low-quality peer relationships, they are likely to encounter additional negative experiences, leading to even lower self-esteem. Considering that both self-esteem and core self-evaluation are intrapersonal attributes, and that a strong association exists between the two (Judge et al., 1998), it is plausible to suggest that peer relationships can significantly moderate the association between parental phubbing and core self-evaluation. Therefore, this study proposes the hypothesis that peer relationships may moderate the association between parental phubbing and core self-evaluation.

### 1.4. The Present Study

**Figure 1:**
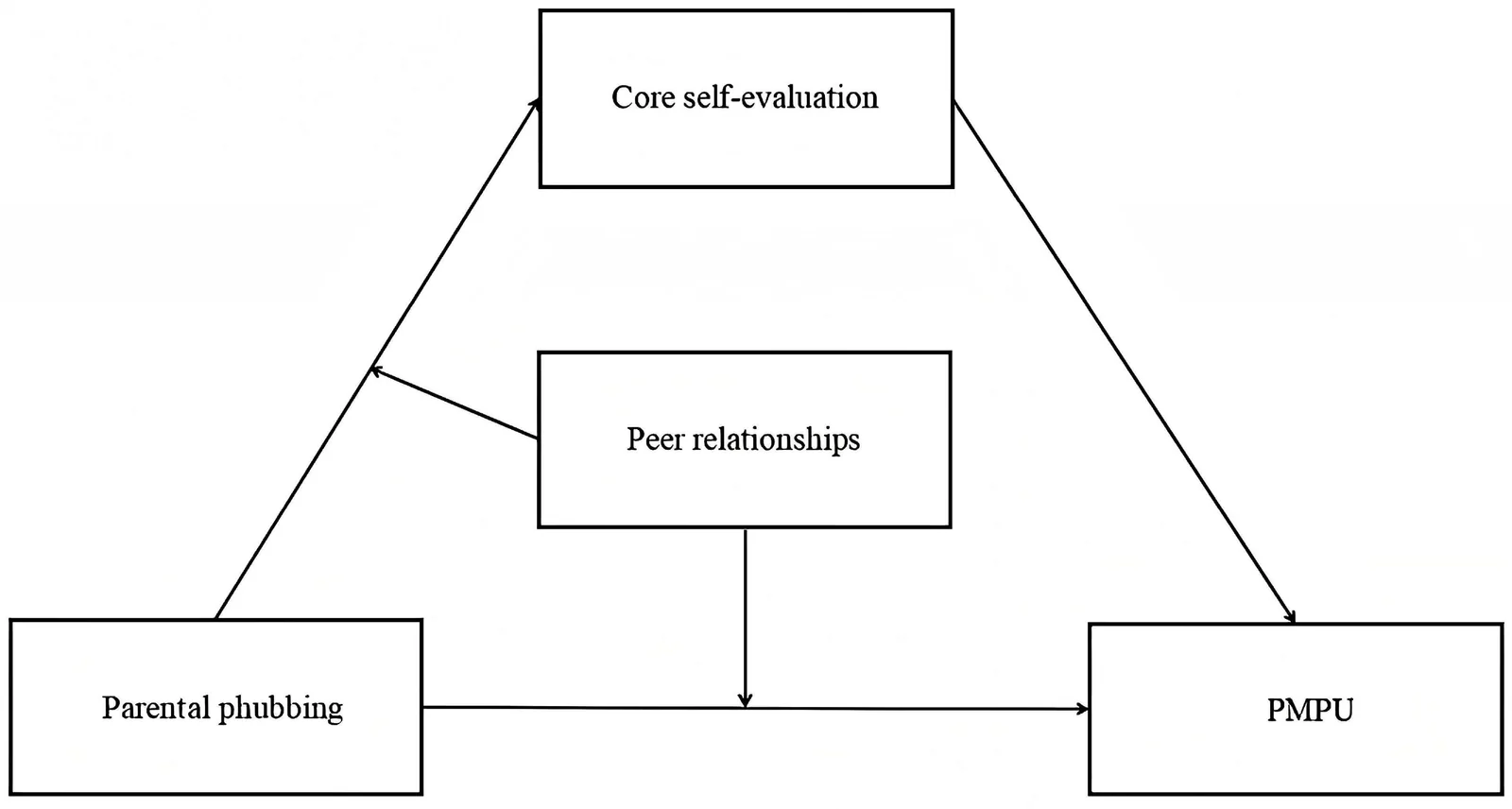
Conceptual Model.

It is important to understand the potential mechanism underlying the association between parental phubbing and PMPU among Chinese adolescents. To this end, the current study used a one-year longitudinal design to clarify the mechanism underlying the association between parental phubbing and PMPU among Chinese adolescents and to test the mediating role of core self-evaluation and the moderating role of peer relationships. Based on the cognitive-behavioral model and ecological systems theory, we proposed the following hypotheses: (1) Parental phubbing is positively associated with adolescent PMPU one year later (Hypothesis 1); (2) Greater parental phubbing is associated with a decrease in core self-evaluation, which in turn is associated with a higher level of PMPU (Hypothesis 2); (3) Peer relationships attenuate the direct association between parental phubbing and PMPU (Hypothesis 3a), as well as the first stage of the mediating process, that is, the association between parental phubbing and core self-evaluation (Hypothesis 3b). The present study makes three incremental contributions beyond existing work. First, although Shen and Xie (2024) established a longitudinal link between parental phubbing and core self-evaluation via basic psychological need satisfaction, the present study is among the first to test peer relationships as a simultaneous moderator of both the direct effect pathway and the first-stage mediation pathway (i.e., a dual moderation structure), thereby revealing when and for whom the mediation mechanism operates most strongly. Second, whereas Mi et al. (2023) examined a conceptually similar moderated mediation model, their study was cross-sectional; the present three-wave longitudinal design provides stronger temporal evidence. Third, the sample of early adolescents (ages 10–14 at baseline) represents a critical developmental window during which both core self-evaluation and PMPU habits are consolidating, making this age group theoretically distinct from the older adolescent samples examined in most prior studies. The proposed conceptual framework is illustrated in Figure 1.

## 2. Materials and Methods

### 2.1. Participants

Participants were recruited from three nine-year compulsory education schools in Guangdong Province, a southern province of China, using a cluster convenience sampling method covering grades 5 to 7 (ages 10 to 14). Data were collected three times, at six-month intervals. At Time 1 (T1, September 2022), 876 adolescents participated in the assessment; at Time 2 (T2, March 2023), 832 students completed the measurements; and at Time 3 (T3, September 2023), 807 adolescents completed the follow-up measurements. In total, 807 adolescents aged 10 to 14 years old participated in all three assessments (M = 12.04 years, SD = 1.44) at T1, and the sample was roughly balanced by gender (384 boys, 423 girls). The rate of sample loss was modest at 5.0% between the first and second time points, and 3.0% between the second and third time points. Attrition analyses comparing the 807 retained adolescents with the 69 who missed at least one later wave revealed no statistically significant disparities on any baseline variable; all between-group differences were negligible (|Cohen’s d| ≤ 0.14; all *p* > 0.29; gender: χ^2^(1) = 0.48, *p* = 0.489), as detailed in Table 1. Importantly, these nonsignificant baseline comparisons and the small effect sizes do not establish that attrition was completely nonsystematic or missing completely at random; attrition may still be related to unmeasured variables or to outcomes that were not observed after dropout. These comparisons should therefore be regarded as descriptive and auxiliary rather than as evidence of random attrition.

### 2.2. Measurements

Importantly, parental phubbing and peer relationships were assessed only at T1 (as distal contextual predictors), core self-evaluation was assessed at T1 and T2, and PMPU was assessed at T1 and T3. This design allowed us to test the hypothesized mediation (T1 PP → T2 CSE → T3 PMPU) while controlling for baseline levels of the mediator and outcome. Cronbach’s alpha values for all scales at each wave are reported below.

#### 2.2.1. Parental Phubbing

The Parental Phubbing Scale, derived from the Partner Phubbing Scale (Roberts & David, 2016) and modified by X. Wang et al. (2020), was utilized to evaluate the adolescent students’ perception of their parents’ phubbing behavior. This scale consists of nine items, comprising statements such as, “During dinner with my parents, they are engrossed in their mobile phones,” and “When we go out together, my parents use their mobile phones.” Respondents rate each item using a five-point Likert scale ranging from 1 (“Never”) to 5 (“All the time”). Afterwards, all item scores were averaged to obtain a total score, with a higher score indicating greater parental phubbing. The modified scale has been validated for use in young people in a Chinese context (K. Liu et al., 2021), and was shown to have robust reliability (Cronbach’s α = 0.91 at T1) in the current study.

#### 2.2.2. Core Self-Evaluation

Core self-evaluation was assessed using the Core Self-Evaluation Scale, as developed by Judge et al. (2003) and later adapted into Chinese by Du et al. (2012). This scale comprises 10 statements, such as, “I am confident I get the success I deserve in life,” and “I am capable of coping with most of my problems.” Respondents indicate their agreement with each statement using a five-point scale ranging from 1 (“Strongly Disagree”) to 5 (“Strongly Agree”). The Chinese version of the scale has been shown to have robust psychometric properties, and has been validated for use within a Chinese cultural context (Du et al., 2012). In the current study, the Cronbach’s α values were 0.89 and 0.88 for T1 and T2, respectively.

#### 2.2.3. Problematic Mobile Phone Use

PMPU was measured using the Mobile Phone Addiction Index, as described by Leung (2008). The scale encompasses 17 items, presented as statements such as, “Your phone usage exceeds your initial intentions,” and “You sacrifice sleep due to extensive phone engagement.” Respondents rate the extent to which each statement applies to them on a five-point scale ranging from 1 (“Never”) to 5 (“Always”). A mean score with a higher value indicates a higher degree of PMPU. The scale has been validated for its effectiveness and dependability among Chinese adolescents (Chen et al., 2021a). In the current study, the Cronbach’s α values were 0.89 at T1 and 0.90 at T3.

#### 2.2.4. Peer Relationships

Participants’ peer relationships were assessed using the Peer Relationship subscale of the Self Description Questionnaire (Marsh et al., 1984). The measure has been shown to have robust validity and reliability when used in adolescent populations in China (Bai et al., 2021). The scale comprises 18 items, such as, “I receive considerable attention from the opposite gender,” and “I can readily form friendships with individuals of the same gender.” Respondents rate each item using a six-point scale ranging from 1 (“Disagree completely”) to 6 (“Agree completely”). A higher score indicates a higher level of peer relationships. In the current study, the Cronbach’s α was 0.86 at T1.

### 2.3. Covariates

Gender, age, and family socioeconomic status (SES) were included as covariates in all structural models, entering each as an exogenous predictor of both T2 core self-evaluation and T3 PMPU. Their inclusion was specified a priori on theoretical grounds (as standard demographic and developmental confounders) rather than decided after inspecting the data. Gender was treated as a dichotomous variable, with males coded as 1 and females as 0. Age was treated as a continuous variable, measured in terms of the adolescent participants’ age in the first semester of 2022. Following a commonly used method in the relevant literature, SES was calculated as the average of participants’ scores across four indicators, each recoded to a common 1–5 metric: family per capita monthly income (10 categories recoded to 1–5), geographical area (5-point scale reflecting urbanization level, 1 = rural to 5 = metropolis), and father’s and mother’s educational levels (each assessed with 8 categories and independently recoded to 1–5). This approach yielded a continuous composite measure of SES ranging from 1 to 5, with higher scores indicating higher SES. Family per capita monthly income was assessed using a 10-category item, where 1 indicated less than or equal to 1000 RMB and 10 indicated more than or equal to 9001 RMB. The adolescent participants were asked to report on the type of geographical area they resided in using a five-point scale reflecting its level of development, ranging from 1 (rural) to 5 (metropolis). The educational level of each parent was measured using an eight-category variable, with 1 representing no formal education and 8 representing a doctoral degree.

### 2.4. Statistical Analysis

The data were analyzed in several steps. First, descriptive statistics and bivariate correlations were computed for the study variables using SPSS 24.0. Second, we assessed whether common method bias was a salient concern, given the use of self-report measures. Third, the hypotheses were tested using observed-variable path analysis in Mplus 8.3 (Muthén & Muthén, 2017), with each construct represented by its scale (mean) composite score; parameters were estimated with the maximum likelihood estimator (ML). To examine Hypotheses 1 and 2, a mediation model was used to examine the mediating effects of T2 core self-evaluation in the association between T1 parental phubbing and T3 PMPU. To examine Hypotheses 3a and 3b, a moderated mediation model was used by adding the moderator (i.e., peer relationships) into the aforementioned mediation model. All structural models adjusted for age, gender, and SES. The two degrees of freedom reported for the path models (df = 2) correspond to two overidentifying restrictions imposed to identify the target longitudinal mediation chain: the path from T1 PMPU to T2 core self-evaluation and the path from T1 core self-evaluation to T3 PMPU are fixed to zero. These restrictions are not claims that the corresponding population associations are exactly zero; they are the minimal constraints needed to identify the focal T1 parental phubbing → T2 core self-evaluation → T3 PMPU chain given the measurement schedule (parental phubbing and peer relationships were assessed only at T1). We explicitly tested these two restrictions in a saturated (full cross-lagged) sensitivity model in which both paths were freely estimated; neither approached statistical significance (β = 0.04 and −0.04, respectively), and the indirect effect was unchanged (Section 3.6.2). More specifically, relative to a saturated recursive model for the same set of observed variables, these two omitted paths are the only overidentifying restrictions: all covariances among the exogenous variables are freely estimated, the two endogenous residual variances are freely estimated, and the residual covariance between the two endogenous variables is fixed to zero as in any recursive model; no other paths or covariances are constrained. The overall model chi-square therefore tests exactly these two restrictions.

Specifically, we tested whether T1 peer relationships moderated the association between T1 parental phubbing and T3 PMPU, and whether T1 peer relationships moderated the association between T1 parental phubbing and T2 core self-evaluation. If an interaction effect was detected, a simple slopes analysis (i.e., 1 SD above and below the mean of the moderator) was performed. Prior to constructing the interaction term, all continuous analysis variables were standardized (M = 0, SD = 1); standardization places the coefficients on a fully standardized metric and reduces nonessential multicollinearity (Aiken & West, 1991). The interaction term was then formed as the product of the standardized parental phubbing and peer-relationship components. Because all continuous variables are standardized before estimation, the reported regression coefficients, conditional indirect effects, and index of moderated mediation are on this standardized metric, and their bias-corrected bootstrap confidence intervals are on the same metric. Two details qualify the standardization terminology. First, Mplus’s STDYX standardization additionally divides the interaction coefficient by the standard deviation of the product term (which is not exactly 1 even when both components are standardized); the interaction coefficients reported throughout are therefore taken from the model estimates based on the product of the standardized components, not from the STDYX block. Second, gender was retained on its original 0/1 scale rather than standardized; the solution is thus standardized with respect to the continuous variables but not with respect to gender. As a robustness check, we also specified a fully latent version in which each construct was represented by three balanced item parcels and the interaction was formed with the matched product-indicator approach; this yielded the same pattern of statistically significant findings; the parcel assignments, fit indices, and path estimates are reported in Section 3.6.1. The longitudinal analyses used the 807 adolescents who completed all three waves; within this retained sample, scale scores were computed as the mean of the completed items when at least two-thirds of a scale’s items had been answered, and were otherwise set to missing; the sparse item-level nonresponse (0.8–1.1% of item data) was thereby incorporated at the scale level, and the remaining scale-level missing values were handled with full information maximum likelihood (FIML; Acock, 2005). Pathway significance was evaluated with a bootstrapping procedure (5000 resamples) using bias-corrected 95% confidence intervals (CI). A 95% CI that did not contain zero indicated a significant path coefficient. To test moderated mediation rigorously, we also estimated the fully standardized conditional indirect effects at low (−1 SD), mean, and high (+1 SD) levels of peer relationships and the fully standardized index of moderated mediation, each with bias-corrected bootstrap 95% CIs (5000 resamples). Following the guidelines proposed by Hoyle (2012), a model was deemed acceptable if the more conservative thresholds of CFI/TLI ≥ 0.95 and RMSEA ≤ 0.06 were met (Hu & Bentler, 1999); the more lenient criteria of χ^2^/df < 5.00, CFI/TLI > 0.90, and RMSEA < 0.08 were also considered acceptable (Hoyle, 2012). The structural models met the more conservative thresholds (CFI/TLI ≥ 0.95, RMSEA ≤ 0.06). For the longitudinal measurement-invariance models, CFI and RMSEA met these thresholds whereas TLI ranged from 0.91 to 0.95; invariance was therefore evaluated using the recommended change-in-CFI (ΔCFI ≤ 0.010) criterion. For clarity, within the primary sample of 807 three-wave completers, FIML operates only on the sparse scale-level missing values arising from item nonresponse (no entire wave is missing for these participants); analyses using all 876 T1 participants, for whom an entire later wave may be missing, are reported only as auxiliary comparisons (e.g., the attrition analysis). The complete Mplus input syntax for the mediation, moderated mediation, measurement-invariance, fully latent, cluster-robust, school fixed-effect, no-covariate, and saturated cross-lagged analyses, together with an annotated output summary, is provided in the Supplementary Materials; the syntax contains no individual-level information.

Prior to estimation we examined the model assumptions. Univariate distributions deviated only slightly from normality (skewness from −0.18 to 0.35; kurtosis from −0.42 to 0.48), which is compatible with maximum likelihood estimation; inference for the indirect effects relied on bias-corrected bootstrap confidence intervals rather than on normality assumptions. Variance inflation factors were small (VIF = 1.08–1.72), indicating no problematic multicollinearity after standardization. Component-plus-residual plots showed no departures from linearity, residuals were approximately normal and homoscedastic, and no observations were unduly influential (maximum Cook’s distance = 0.34).

## 3. Results

### 3.1. Common Method Bias

Because the survey responses were provided solely by adolescents, we evaluated common method bias using Harman’s one-factor test: all questionnaire items were entered into an unrotated principal component analysis in SPSS 24.0, as recommended by Podsakoff et al. (2003). This test revealed seven factors with eigenvalues exceeding 1, without applying rotation. The initial factor accounted for 17.79% of the variance. Because a first factor above approximately 40% is commonly taken to indicate substantial common method bias, these results provided no strong evidence of substantial bias. We note, however, that Harman’s one-factor test is insensitive and, on its own, cannot rule out common method bias; we therefore interpret this result cautiously and complement it with the longitudinal design (see Section 5).

### 3.2. Descriptive Statistics

Table 2 presents the mean values and standard deviations across all variables, as well as their mutual correlations. Parental phubbing at T1 was positively correlated with PMPU at T3, suggesting that parental phubbing may be a risk factor for later adolescent PMPU. Conversely, parental phubbing at T1 was found to have a negative association with core self-evaluation assessed at T2, suggesting that greater parental phubbing is associated with lower core self-evaluation in adolescents. Moreover, a negative relationship was found between core self-evaluation at T2 and PMPU at T3, indicating that higher core self-evaluation is associated with lower PMPU. On average, PMPU increased significantly from T1 (M = 3.63, SD = 0.87) to T3 (M = 3.82, SD = 0.90), t(806) = 4.78, *p* < 0.001, Cohen’s d = 0.17, indicating a modest normative upward trend.

### 3.3. Measurement Models and Longitudinal Measurement Invariance

Because Cronbach’s alpha alone does not establish construct validity or temporal comparability, we evaluated the measurement models and tested longitudinal measurement invariance. Each longitudinal construct was represented by four balanced item parcels (as opposed to the three parcels used for the fully latent robustness check in Section 2.4). Parcels were built with a loadings-balanced procedure, assigning items within each construct so that the parcels contained comparable numbers of items and approximately equal average factor loadings (in the four-parcel models, 2–3 items per parcel for the 10-item core self-evaluation scale and 4–5 for the 17-item PMPU scale). The four-parcel allocation was used for the invariance tests because it keeps the parcels balanced and avoids the uneven 4/3/3 split that three parcels would impose on the 10-item scale; the three-parcel allocation was reserved for the latent robustness check (Section 2.4) to match the established matched product-indicator specification. We estimated configural, metric, and scalar models, comparing nested models by change in CFI (ΔCFI ≤ 0.010 supporting invariance; Cheung & Rensvold, 2002) and RMSEA. Single-wave measurement models fit well: CSE T1, χ^2^(2) = 5.8, CFI = 0.994, RMSEA = 0.049; CSE T2, χ^2^(2) = 5.1, CFI = 0.995, RMSEA = 0.044; PMPU T1, χ^2^(2) = 7.2, CFI = 0.990, RMSEA = 0.057; PMPU T3, χ^2^(2) = 6.6, CFI = 0.991, RMSEA = 0.054. The exact four-parcel assignments are listed in Appendix A, Table A2. We note that these parcel-level invariance tests support the temporal comparability of the parceled scores but do not establish item-level measurement invariance or item-level construct validity (see Section 3.6.1 and Section 5).

As shown in Table 3, for both core self-evaluation (T1–T2) and PMPU (T1–T3), metric and scalar invariance were supported (all ΔCFI ≤ 0.007), indicating that the constructs retained equivalent meaning over time and that longitudinal coefficients and mean levels can validly be compared.

### 3.4. Testing the Mediation Model

**Figure 2:**
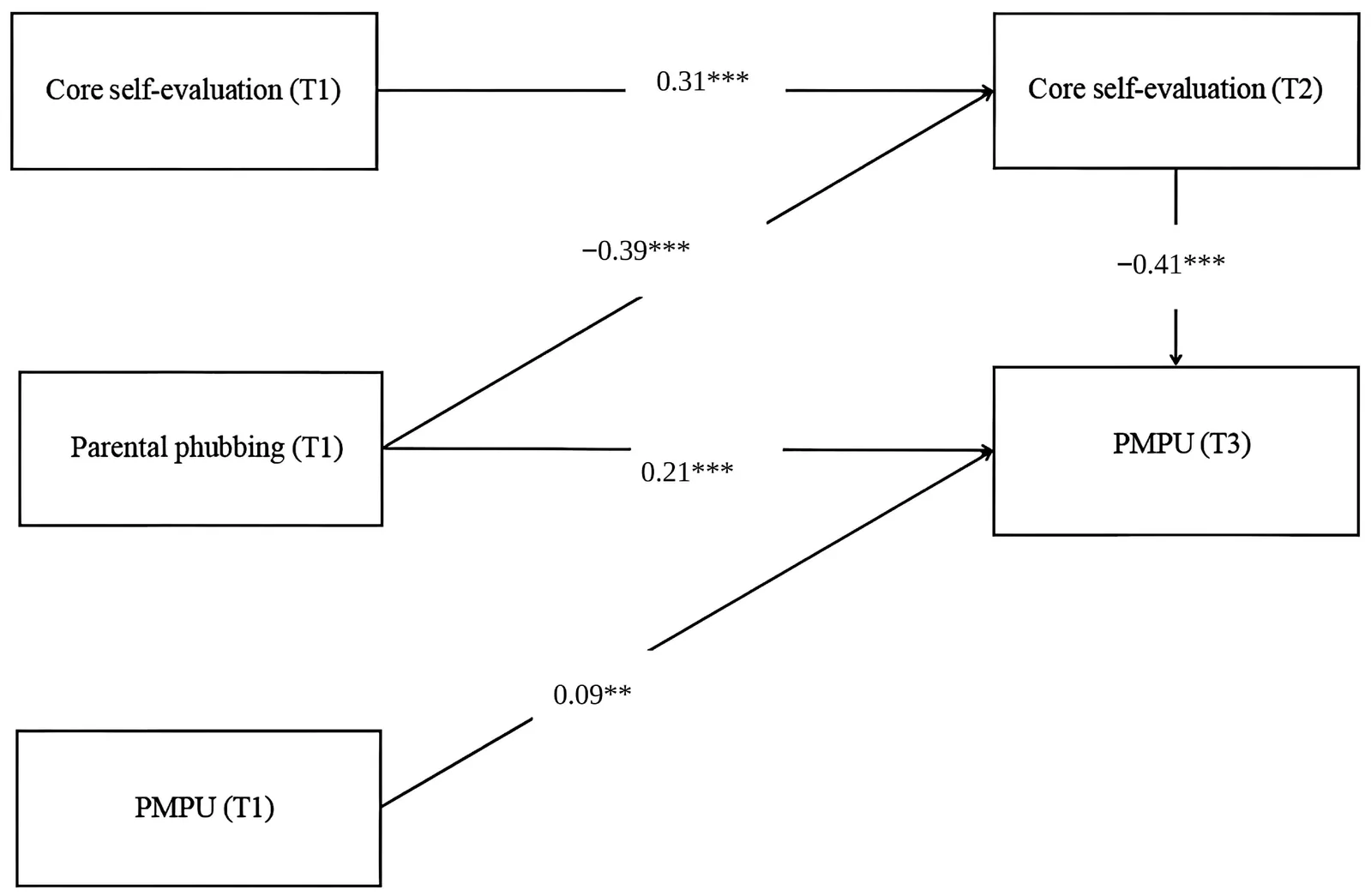
Structural model of the mediating role of core self-evaluation at Time 2 (T2) between parental phubbing at Time 1 (T1) and PMPU at Time 3 (T3). Path coefficients are standardized regression coefficients from the simple mediation model. Baseline measures (T1 core self-evaluation and T1 PMPU) and covariates (age, gender, and SES) were included in the model; the complete model specification is available in the Mplus syntax provided in the Supplementary Materials. ** *p* < 0.01, *** *p* < 0.001.

In the simple mediation model, a positive direct effect was found between parental phubbing at T1 and PMPU at T3 (β = 0.21, *p* < 0.001, 95% CI = [0.14, 0.28]), controlling for baseline PMPU at T1. All estimates adjusted for age, gender, and SES. Regarding the covariates, family SES positively predicted T2 core self-evaluation (β = 0.30, *p* < 0.001), whereas its association with T3 PMPU and the gender and age effects were non-significant. The mediation model depicted in Figure 2 demonstrated a suitable fit to the data, with indices as follows: χ^2^(2) = 5.21, χ^2^/df = 2.61, *p* = 0.074, CFI = 0.995, TLI = 0.970, SRMR = 0.017, and RMSEA = 0.045 [90% CI: 0.000, 0.094]. The relatively wide RMSEA confidence interval is a known statistical artifact in models with very few degrees of freedom (Kenny et al., 2015), and should not be interpreted as indicating poor fit, given that all other indices (CFI, TLI, SRMR) exceeded recommended thresholds. Meanwhile, a negative association was revealed between parental phubbing at T1 and core self-evaluation at T2 (β = −0.39, *p* < 0.001, 95% CI = [−0.45, −0.33]; the corresponding conditional path at the mean of peer relationships is −0.38 in the moderated mediation model that adds the interaction terms, Section 3.5), controlling for baseline core self-evaluation at T1. Additionally, core self-evaluation at T2 was negatively associated with PMPU at T3 (β = −0.41, *p* < 0.001, 95% CI = [−0.47, −0.35]), controlling for baseline PMPU at T1. Bootstrap analysis confirmed that core self-evaluation at Time 2 significantly mediated the longitudinal association between parental phubbing at T1 and PMPU at T3 (indirect effect = 0.16, 95% bias-corrected bootstrap CI = [0.12, 0.21]). The autoregressive paths were also significant: T1 core self-evaluation predicted T2 core self-evaluation (β = 0.31, *p* < 0.001) and T1 PMPU predicted T3 PMPU (β = 0.09, *p* < 0.01).

### 3.5. Testing the Moderated Mediation Model

**Figure 3:**
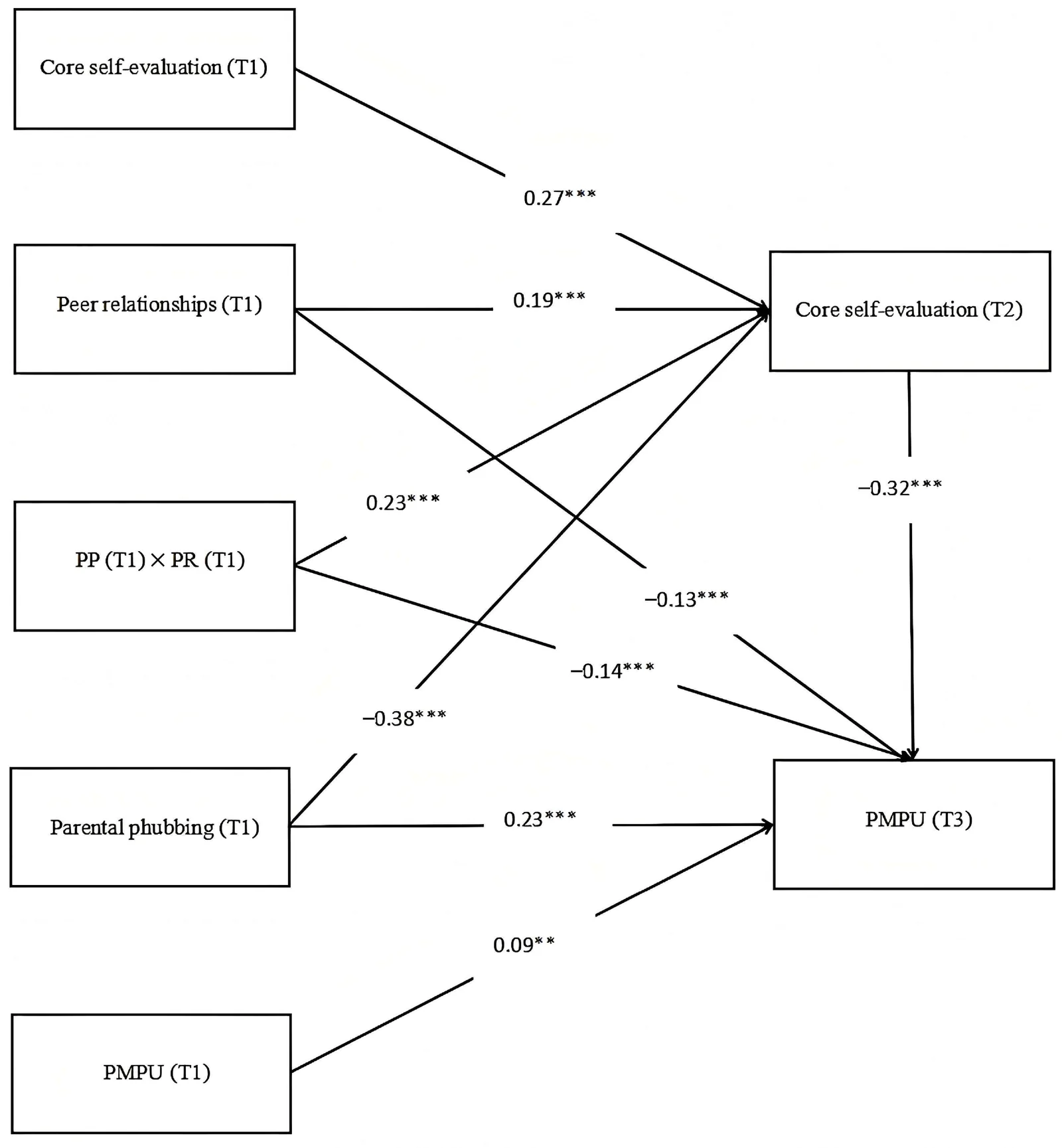
The moderated mediation model involving peer relationships at Time 1 (T1) and core self-evaluation at Time 2 (T2) in the association between parental phubbing at T1 and PMPU at Time 3 (T3). Path coefficients are standardized regression coefficients. Baseline measures (T1 core self-evaluation and T1 PMPU) and covariates (age, gender, and SES) were included in the model; the complete model specification is available in the Mplus syntax provided in the Supplementary Materials. ** *p* < 0.01, *** *p* < 0.001.

Building on the established mediation framework, we tested a moderated mediation model (Figure 3) to evaluate whether peer relationships buffer the adverse effects of parental phubbing. The model exhibited an excellent fit to the data: χ^2^(2) = 3.45, χ^2^/df = 1.72, *p* = 0.178, CFI = 0.998, TLI = 0.984, SRMR = 0.013, and RMSEA = 0.030, explaining 48% of the variance in T2 core self-evaluation (R^2^ = 0.48) and 35% of the variance in T3 PMPU (R^2^ = 0.35).

**Figure 4:**
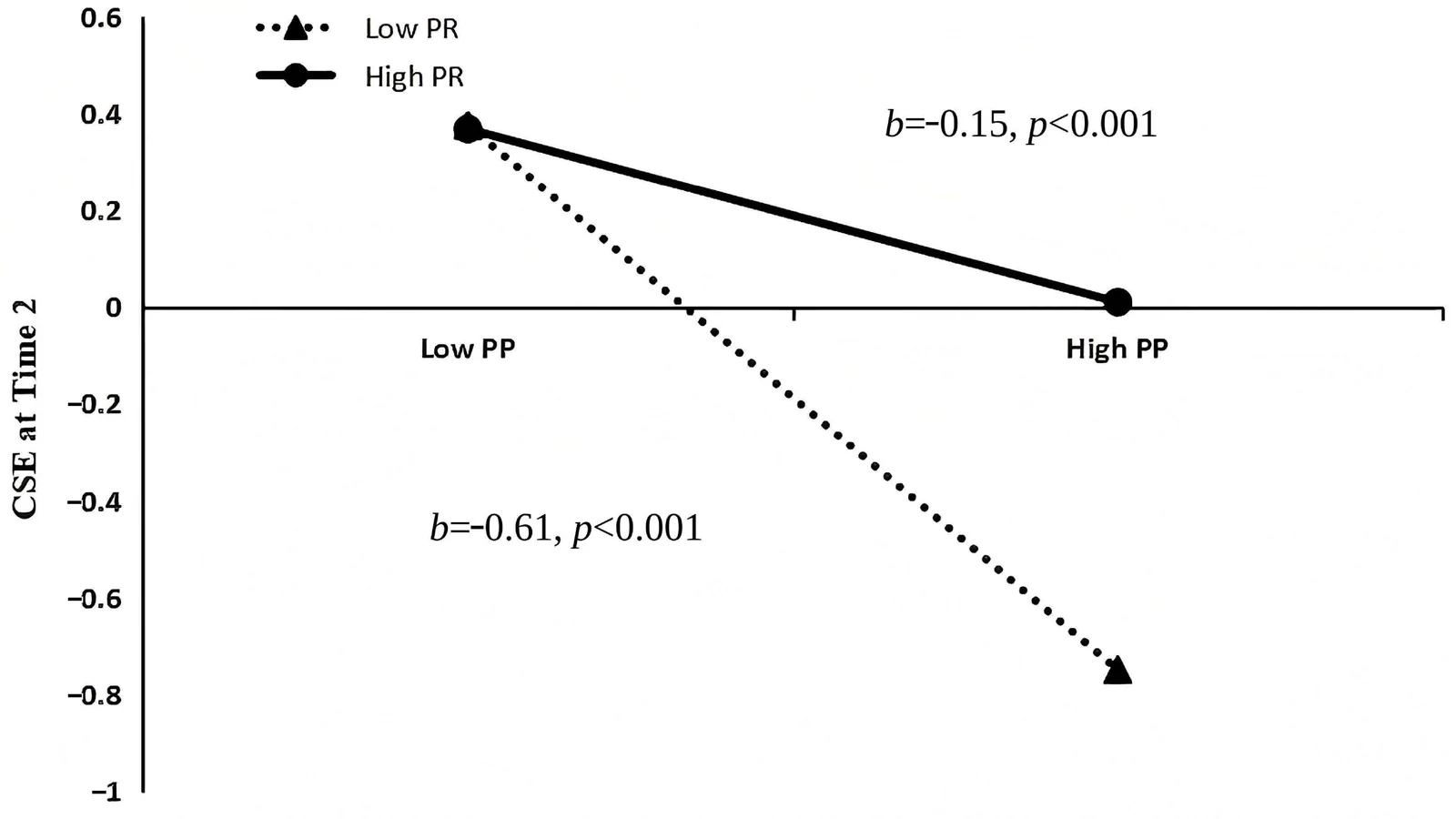
The relationship between parental phubbing at Time 1 and core self-evaluation (CSE) at Time 2 moderated by peer relationships at Time 1. PP = parental phubbing; PR = peer relationships. High PR and Low PR represent ±1 SD from the mean of peer relationships. All variables are standardized.

**Figure 5:**
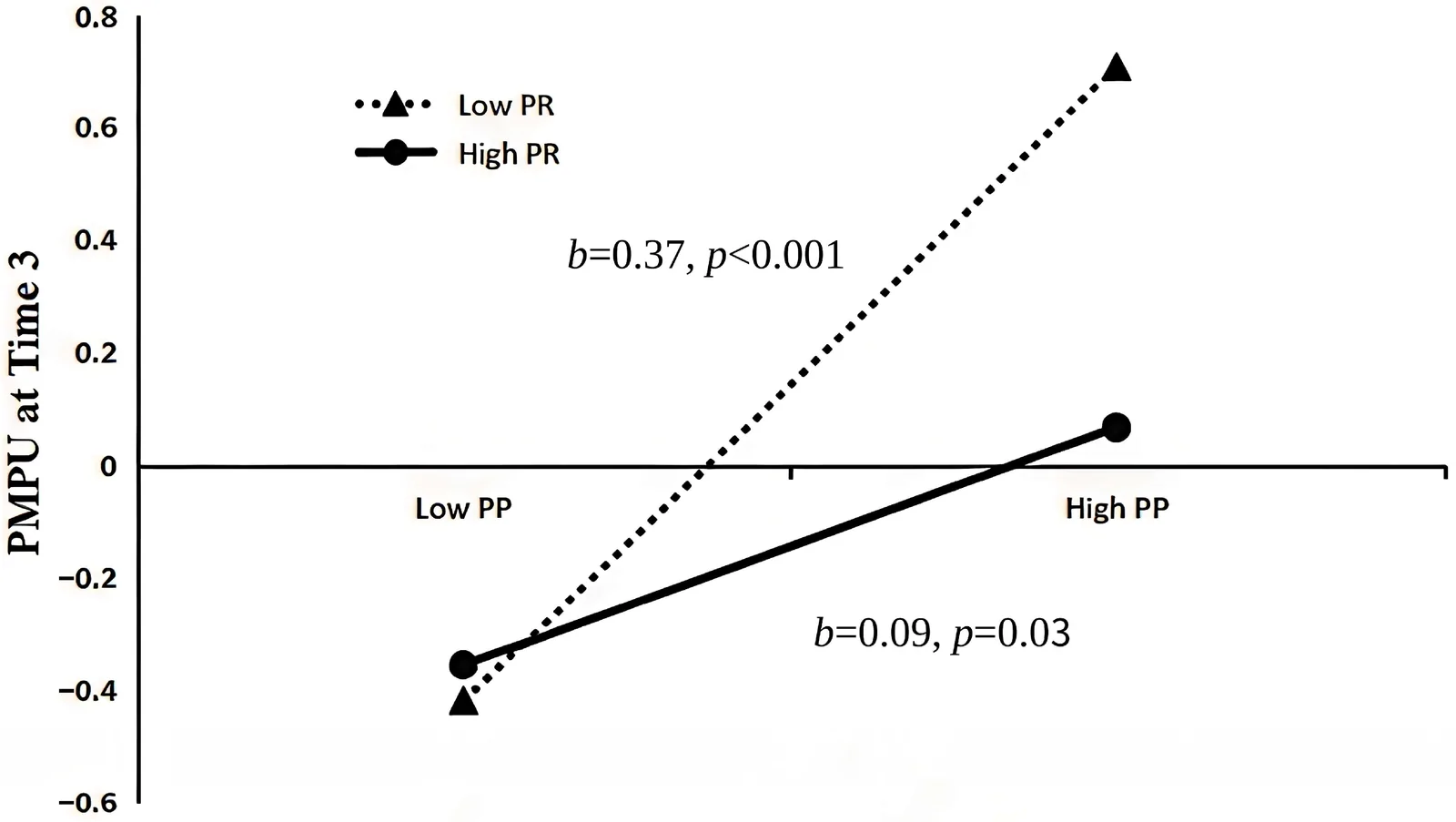
The relationship between parental phubbing at Time 1 and PMPU at Time 3 moderated by peer relationships at Time 1. PP = parental phubbing; PR = peer relationships. High PR and Low PR represent ±1 SD from the mean of peer relationships. All variables are standardized.

As hypothesized, peer relationships significantly moderated both the path from parental phubbing to core self-evaluation (β = 0.23, *p* < 0.001, 95% CI = [0.18, 0.28]) and the direct path from parental phubbing to PMPU (β = −0.14, *p* < 0.001, 95% CI = [−0.19, −0.08]), controlling for baseline scores. Simple slopes analyses were conducted to probe these interactions (±1 SD). As shown in Figure 4, the negative association between parental phubbing and core self-evaluation was significantly weaker for adolescents with high peer relationships (β = −0.15, *p* < 0.001, 95% CI = [−0.22, −0.08]) compared to those with low peer relationships (β = −0.61, *p* < 0.001, 95% CI = [−0.68, −0.55]). Similarly, Figure 5 illustrates that the positive association between parental phubbing and PMPU was attenuated for adolescents with high peer relationships (β = 0.09, *p* = 0.03, 95% CI = [0.01, 0.18]) relative to those with low peer relationships (β = 0.37, *p* < 0.001, 95% CI = [0.29, 0.45]). In this moderated mediation model, the conditional direct effect of parental phubbing on PMPU at the mean level of peer relationships was β = 0.23 (*p* < 0.001, 95% CI = [0.17, 0.30]). This value corresponds to the path coefficient shown in Figure 3. For completeness, T1 peer relationships also showed a positive main association with T2 core self-evaluation (β = 0.19, *p* < 0.001) and a negative main association with T3 PMPU (β = −0.13, *p* < 0.001). The core self-evaluation autoregressive path in this moderated model was β = 0.27 (*p* < 0.001), slightly below the 0.31 estimate in the simple mediation model because peer relationships and the interaction terms were added. Crucially, supporting the central moderated mediation hypothesis, the conditional indirect effect of T1 parental phubbing on T3 PMPU (via T2 core self-evaluation) varied systematically with peer relationships (all on the fully standardized metric; Section 2.4): it was 0.20 (95% bootstrap CI [0.15, 0.24]) at low PR, 0.12 ([0.10, 0.15]) at mean PR, and 0.05 ([0.03, 0.07]) at high PR (Table 4). The index of moderated mediation was −0.074 (95% bootstrap CI [−0.097, −0.054]), confirming that the indirect effect was significantly weaker at higher peer-relationship levels. No M × W (T2 CSE × T1 peer relationships) interaction was included; the T2 CSE to T3 PMPU (b) path was therefore held invariant across peer-relationship levels, on the grounds that peer support was expected to buffer the family-to-self and family-to-behavior links rather than the self-to-behavior link. Accordingly, the index of moderated mediation (a1*b = −0.074) captures first-stage moderation only, whereas the X × W to Y coefficient (−0.14) separately moderates the direct effect c-prime and is not part of the indirect path.

### 3.6. Robustness Checks: School Clustering and Covariate Adjustment

Participants were nested within three schools (T1: School A = 318, School B = 296, School C = 262; T2: 302, 281, 249; T3: 293, 273, 241, respectively). We first re-estimated the moderated mediation with cluster-robust standard errors (Mplus TYPE = COMPLEX); because only three clusters are available (too few for the correction to be fully reliable), we also fit a model with school as a fixed effect. Under cluster-robust estimation the point estimates were unchanged and intervals slightly wider, yet all hypothesized paths remained significant (Table 5). The fixed-effect model gave virtually identical estimates (a = −0.38, b = −0.31, c′ = 0.22, X × PR→M = 0.22, X × PR→Y = −0.13; mean-PR indirect = 0.12), all significant. Finally, comparing the primary (covariate-adjusted) models with otherwise identical models omitting age, gender, and SES left the effects essentially unchanged (a: −0.38 vs. −0.40; b: −0.32 vs. −0.33; c′: 0.23 vs. 0.25; indirect: 0.12 vs. 0.13; X × PR→M: 0.23 vs. 0.24; X × PR→Y: −0.14 vs. −0.15). The corresponding Mplus inputs and outputs for the school fixed-effect and no-covariate specifications are provided as Syntax 6 and Syntax 7 in the Supplementary Materials. These analyses should be regarded as exploratory sensitivity checks rather than as evidence that clustering-related inferential concerns are resolved: the point estimates were stable and covariate adjustment had little effect, but with only three schools the cluster-robust correction cannot be treated as reliable, and the school fixed effects control only between-school mean differences. The mediation and moderation point estimates were stable to these specifications and to covariate adjustment, while we acknowledge the inherent limitation of only three schools. For completeness, the TYPE = COMPLEX sensitivity analysis uses Mplus’s sandwich (robust) standard errors (MLR within TYPE = COMPLEX); it is a separate sensitivity specification from the primary ML models and is not part of the bootstrap-based inference.

#### 3.6.1. Fully Latent Robustness Analysis

In the fully latent robustness model, each construct was represented by three balanced item parcels, and the latent interaction was formed with the matched product-indicator approach (matching parcels by their rank-ordered loadings). Parcels were assigned with a loadings-balanced procedure so that each parcel contained a comparable number of items and approximately equal average loadings (the 9-item parental phubbing scale was split 3/3/3; the 10-item core self-evaluation scale 4/3/3; the 17-item PMPU scale 6/6/5; and the 18-item peer-relationship scale 6/6/6). The model fit the data adequately: χ^2^(175) = 278.2, CFI = 0.971, TLI = 0.965, RMSEA = 0.027 [90% CI: 0.021, 0.033], SRMR = 0.043. The principal estimates were consistent with the observed-variable results: a = −0.41 (95% CI [−0.49, −0.33]), b = −0.34 ([−0.42, −0.26]), the mean-level c′ = 0.24 ([0.15, 0.33]), X × PR→M = 0.24 ([0.16, 0.32]), and X × PR→Y = −0.15 ([−0.23, −0.07]). The conditional indirect effects were 0.22 ([0.16, 0.29]) at low PR, 0.14 ([0.10, 0.19]) at mean PR, and 0.06 ([0.02, 0.11]) at high PR, with an index of moderated mediation of −0.082 ([−0.118, −0.050]). The exact three-parcel assignments are listed in Appendix A, Table A1. This latent verification specification did not include the age, gender, and SES covariates; the corresponding observed-variable models that adjust for these covariates are reported above.

We note that the parcel-level measurement invariance and these parcel-based latent results support the temporal comparability of the parceled constructs but do not establish item-level measurement invariance or item-level construct validity; item-level analyses with additional waves remain for future research.

#### 3.6.2. Saturated Cross-Lagged Sensitivity Model

To directly test the two overidentifying restrictions of the primary path models (Section 2.4), we fitted a saturated (full cross-lagged) model in which both restricted paths were freely estimated: T1 PMPU → T2 core self-evaluation and T1 core self-evaluation → T3 PMPU. As expected for a saturated recursive model, this model had zero chi-square degrees of freedom. Neither freed path approached statistical significance (T1 PMPU → T2 CSE: β = 0.04, *p* = 0.14; T1 CSE → T3 PMPU: β = −0.04, *p* = 0.24), and the remaining estimates were virtually unchanged (a = −0.40, b = −0.39, c′ = 0.21; autoregressive paths 0.31 and 0.09). Most importantly, the indirect effect remained 0.16 with an unchanged bias-corrected bootstrap 95% CI [0.12, 0.21]. The target mediation chain is therefore robust to relaxing both restrictions; the corresponding input and output are provided as Syntax 8 in the Supplementary Materials.

## 4. Discussion

This study examined the relationship between parental phubbing and PMPU from two aspects: environmental factors (i.e., parental phubbing, peer relationships) and individual factors (i.e., core self-evaluation). Guided by the cognitive-behavioral model and ecological systems theory, this study adopted a one-year longitudinal design to examine the longitudinal associations between parental phubbing and PMPU. Core self-evaluation was found to significantly mediate the longitudinal association between parental phubbing and adolescent PMPU, with the conditional direct effect of parental phubbing on PMPU varying as a function of peer relationships. In addition, peer relationships were found to play a positive moderating role. Specifically, among adolescents with higher peer relationships, the negative association of parental phubbing with core self-evaluation was weaker, core self-evaluation was higher, and the risk of PMPU was lower.

### 4.1. Parental Phubbing and PMPU

The findings of this study reveal that parental phubbing was significantly associated with higher levels of PMPU among adolescents 12 months later, which aligns with and extends the results of previous cross-sectional studies (Xie et al., 2019; Niu et al., 2020). One plausible explanation for this association is that parental phubbing disrupts effective communication within the family, creating a void in the fulfillment of adolescents’ psychological needs such as competence, affiliation, and autonomy (Gutman & Eccles, 2007). When these needs are not met in their immediate environment, adolescents may turn to the Internet, particularly through their mobile phones, as a source of comfort and validation. This aligns with stage-environment fit theory, which emphasizes the importance of a supportive family environment in meeting adolescents’ psychological necessities for healthy development. Furthermore, the intergenerational transmission effect provides another lens through which to understand this relationship. Adolescents often unconsciously imitate their parents’ behaviors and attitudes, and this includes the parents’ mobile phone use (Niu et al., 2020). When parents prioritize their devices over interactions with their children, they set a precedent that may encourage similar behaviors in adolescents. The results of this one-year longitudinal study contribute to the existing literature by providing evidence consistent with a prospective association between parental phubbing and PMPU that persisted across the one-year period, and they point to the potential value of prevention efforts that involve both parents and adolescents. The concurrent association between T1 parental phubbing and T1 PMPU was moderate in magnitude (r = 0.30, *p* < 0.001), and the one-year longitudinal association was somewhat stronger (r = 0.42, *p* < 0.001). This pattern is consistent with a cumulative-risk model of family dysfunction (Repetti et al., 2002), according to which the behavioral implications of adverse family environments strengthen as exposure is prolonged. Adolescents may initially draw on other coping resources to offset perceived parental neglect, but sustained and repeated exposure can gradually erode these resources, with problematic phone use emerging later. As Cole and Maxwell (2003) note, longitudinal designs are especially informative for detecting such time-dependent processes. We further note that the one-year stability of PMPU was modest (r = 0.19), consistent with the still-crystallizing, partly state-like nature of phone-use patterns in early adolescence (see Section 4.2).

### 4.2. The Mediating Role of Core Self-Evaluation in the Phubbing–PMPU Link

The results of this study also support the mediating role of core self-evaluation on the link between parental phubbing and adolescent PMPU. Specifically, adolescents with lower levels of core self-evaluation are more susceptible to PMPU when exposed to elevated levels of parental phubbing. This finding supports Hypothesis 2 and further substantiates Davis’ cognitive-behavioral model. When parents exhibit adverse parenting behaviors, adolescents tend to develop more negative cognitive patterns, potentially leading them to perceive themselves more negatively (e.g., “It’s all my fault,” or “I’m not good enough”; Geng et al., 2021; Zhang et al., 2021). Such dysfunctional thinking leads to a disinclination to engage in effortful pursuits in reality, prompting the adoption of avoidant coping strategies (Gong et al., 2023). Mobile phones, with their instant feedback and tolerance for setbacks (Li et al., 2022), emerge as a readily available and appealing escape route, especially for those with low self-worth, increasing their likelihood of PMPU. Based on this theory, parental phubbing behavior, as a stressful life event occurring in the family environment, may constitute an important distal factor in the model, while core self-evaluation, as a crucial cognitive factor for individuals, may serve as a key proximal factor. That is, parental phubbing behavior may lead adolescents to develop maladaptive cognitions, such as low core self-evaluation, which in turn may result in the behavioral symptom of PMPU. Understanding this, therefore, the findings may inform the design of future prevention strategies for adolescent PMPU, whose effectiveness must be established empirically. Approaches that foster adolescents’ core self-evaluation (for example, schema- or narrative-oriented work that future intervention research could evaluate) may help reduce the negative effects of challenging family environments on adolescents’ psychological maturation and, potentially, their likelihood of PMPU; the present study, however, did not test any such intervention (Langroudi et al., 2011). It is worth noting that the correlation between T1 core self-evaluation and SES was negligible (r = −0.01), whereas T2 core self-evaluation showed a positive association with SES (r = 0.29). This discrepancy may reflect developmental changes in how adolescents perceive their family’s social standing: early adolescents may not yet fully internalize SES-related social comparisons, but as they mature and become more socially aware, family resources increasingly shape their self-evaluations (Robins & Trzesniewski, 2005). Additionally, the strong correlation between T2 core self-evaluation and T3 PMPU (r = −0.52) is consistent with the cognitive-behavioral model’s prediction that maladaptive cognitions serve as proximal predictors of addictive behaviors (Davis, 2001), and the 6-month interval may have captured a period of heightened vulnerability during which diminished self-evaluation translates more directly into compulsive phone use.

An additional developmental observation concerns the relatively low stability of PMPU across the one-year interval. The mean level of PMPU showed a modest but statistically significant increase from T1 (M = 3.63, SD = 0.87) to T3 (M = 3.82, SD = 0.90), t(806) = 4.78, *p* < 0.001, Cohen’s d = 0.17, suggesting a normative developmental trend of increasing phone-related problems during early adolescence. The low autoregressive coefficient (β = 0.09) combined with this mean-level increase indicates that PMPU during early adolescence is characterized by substantial rank-order instability alongside a general upward trajectory. By comparison, core self-evaluation showed a higher zero-order six-month test–retest correlation (T1–T2, r = 0.40), indicating somewhat greater, though still only moderate, rank-order stability. This pattern is consistent with the view that problematic phone use in this age group is more state-like than trait-like, being highly responsive to contextual factors such as changing peer norms, evolving phone access, and fluctuating family dynamics. This developmental instability has important implications: it suggests that early adolescence represents a window of opportunity for intervention, as problematic use patterns have not yet crystallized into stable habits.

### 4.3. The Moderating Role of Peer Relationships

This study also revealed that peer relationships moderated the association between parental phubbing and PMPU. More precisely, peer relationships attenuated both the direct association of parental phubbing with PMPU (Hypothesis 3a) and the first stage of the mediating association of parental phubbing with core self-evaluation (Hypothesis 3b). Peer relationships acted as a moderator: among adolescents with high-quality peer relationships, the predictive strength of parental phubbing for later PMPU was attenuated. This could be due to several reasons. First, peer relationships are an important source of social support for adolescents and can compensate for a lack of family support, attenuating the association between a negative family environment and adolescent PMPU (Huang et al., 2022). Second, good peer relationships help to improve an adolescent’s sense of group acceptance and belonging (P. Wang et al., 2017), thereby reducing their likelihood of developing PMPU. Conversely, adolescents with poor peer relationships are more prone to feelings of loneliness and helplessness (Duman & Ozkara, 2019; Xiang et al., 2023). When adolescents who experience parental phubbing simultaneously have unhealthy peer relationships, the dual pressures from both family and society can leave their needs significantly unmet in the real-world environment (Costa et al., 2016). This, in turn, drives these individuals to engage further in avoidance behaviors, turning to the Internet as a source of solace and validation, and consequently increasing their risk of PMPU.

This study also found that peer relationships moderated the association between parental phubbing and adolescents’ core self-evaluation. Specifically, poor peer relationships strengthened the negative association of parental phubbing with individuals’ core self-evaluations, whereas this negative predictive effect was less pronounced among adolescents with good peer relationships. Individuals with higher-quality peer relationships are more likely to believe that their relationship with their peers is close and unbreakable, and that they can turn to their peers for support, thus compensating for a lack of security and belonging that may be associated with parental phubbing in their home environment. As these individuals believe that they occupy an irreplaceable position in the hearts of their friends, this contributes to a higher level of core self-evaluation (Huang et al., 2022). In contrast, adolescents with low-quality peer relationships feel that their relationships with their peers are distant or unsatisfying, which then aggravates the sense of helplessness and loneliness associated with their experience of parental phubbing, leading them to believe they are worthless in the eyes of their friends and family, thus making them more likely to lower their evaluation of themselves (H. Wang et al., 2023). These results provide further support for the ecological systems theory (Bronfenbrenner, 1979), in that the development of one’s emotions, cognition, behavior, and other aspects are shaped by the interplay of the behaviors of significant others in one’s individual micro-system. Parents and peers fulfill significant functions in an adolescent’s micro-system, and parental phubbing and the quality of one’s peer relationships become effective evaluative indicators of parents and peers, both of which interact and are jointly associated with an individual’s cognition (i.e., core self-evaluation) and behaviors (PMPU). To address adolescents’ cognitive (core self-evaluation) and behavioral (PMPU) issues, future prevention efforts might target both the familial and social environments. At the family level, parents may be encouraged to reduce their phubbing behavior and engage in more face-to-face interactions with their children, fostering a more supportive and connected family atmosphere. At the social level, schools and communities could explore promoting healthy peer relationships (for instance, through carefully evaluated team-building or social-skills programs) that may provide adolescents with a sense of belonging and group acceptance; such programs remain to be empirically tested. Coordinated efforts across the family and peer microsystems have been proposed as one way to support adolescent well-being; whether such efforts effectively improve outcomes remains to be established through dedicated intervention trials, as the present study did not test any intervention.

This buffering pattern is consistent with recent evidence that the pathways linking adverse experiences to later adjustment are carried by individual-level resources: positive humor styles and tolerance of ambiguity, for example, serially mediate the pathway from childhood adversity to resilience (Suriano & Valentino, 2026). These specific mechanisms, however, were not measured in the present study.

The present findings should also be interpreted within the context of Chinese cultural values, which may shape the relationship between parental phubbing and adolescent PMPU in ways distinct from Western samples. First, the Confucian emphasis on filial piety and parental authority may intensify adolescents’ interpretation of parental phubbing as a form of relational rejection. In Chinese families, parental attention and engagement are traditionally viewed as expressions of care and moral obligation; when parents divert attention to their phones, adolescents may perceive this as a more severe violation of expected family dynamics than their Western counterparts would (X. Wang et al., 2022). Second, Chinese adolescents face extraordinary academic pressure, particularly in the context of the gaokao-oriented education system. Mobile phones represent both a tempting escape from academic stress and a source of conflict with parents who view phone use as interfering with study. This tension may amplify the association between parental phubbing and PMPU, as adolescents who perceive their parents as disengaged may feel less obligated to comply with parental expectations regarding phone use. Third, in collectivist cultures, peer relationships may serve as a more powerful buffer against family dysfunction than in individualistic contexts, because peer groups in China often function as a surrogate family providing emotional support, normative guidance, and a sense of belonging that compensates for deficits in the family microsystem (P. Wang et al., 2017). The significant moderating role of peer relationships observed in this study is consistent with this interpretation. Future cross-cultural studies should directly test whether these cultural mechanisms account for differences in the parental phubbing–PMPU link between Chinese and Western adolescent samples. Because none of these cultural mechanisms were directly measured here and no cross-cultural comparison was conducted, the preceding cultural accounts are offered as plausible contextual interpretations rather than empirically demonstrated mechanisms.

## 5. Limitations and Future Directions

First, all variables were assessed via self-report, which introduces the potential for common method bias. Although Harman’s one-factor test provided no strong evidence of dominant common method variance (first factor accounting for 17.79%), this method has been criticized for its low sensitivity (Podsakoff et al., 2003). However, the longitudinal design with 6- and 12-month intervals substantially reduces the likelihood that common method variance inflated the observed associations compared to cross-sectional designs (Podsakoff et al., 2003; Cole & Maxwell, 2003). Consistent with this reasoning, our key tests concern cross-wave associations, which are less vulnerable to same-wave method inflation. We note that the concurrent correlation between T1 parental phubbing and T1 PMPU was moderate (r = 0.30, *p* < 0.001); because both constructs were self-reported at the same wave, this value may itself partly reflect shared method variance, and we therefore base our conclusions on the longitudinal rather than the concurrent associations. Nevertheless, future studies should employ multi-informant designs (e.g., parent reports of phubbing, objective screen-time data) and consider unmeasured latent method construct (ULMC) analyses to more rigorously address this limitation. Additionally, the current study did not distinguish between fathers and mothers; given that paternal and maternal phubbing may have distinct effects on adolescent adjustment, future research should examine parent-specific influences.

Second, the restriction of the current sample to early adolescents from three schools in Guangdong Province limits the generalizability of the findings to broader Chinese adolescent populations, let alone cross-cultural contexts. Given that perceived parental phubbing may be associated with mental health and behavior differently in collectivist societies such as China as compared to in individualistic Western societies (X. Wang et al., 2022), broader, large-scale population studies are warranted to strengthen the generalizability of the findings.

Third, the analytical approach has two notable constraints. On one hand, the current model employed a single-path longitudinal mediation design (T1 predictor → T2 mediator → T3 outcome) without simultaneously estimating full autoregressive paths for all endogenous variables. This is partly a consequence of the measurement schedule: parental phubbing and peer relationships were assessed only at T1, precluding full cross-lagged estimation. Future research should measure all constructs at every wave and employ a cross-lagged panel model (CLPM) or, ideally, a random-intercept cross-lagged panel model (RI-CLPM) to disentangle between-person stability from within-person change. On the other hand, participants were recruited from only three schools, creating a nested data structure. We therefore conducted exploratory cluster-robust (TYPE = COMPLEX) and school fixed-effect sensitivity analyses, in which the effects remained essentially unchanged and significant, although these corrections are themselves limited by having only three clusters; the small cluster count could nonetheless yield underestimated standard errors and inflated Type I error rates. Future studies with larger numbers of schools should employ multilevel modeling or design-based standard error corrections.

Fourth, although we tested longitudinal measurement invariance separately for core self-evaluation (T1–T2) and PMPU (T1–T3) and found support for metric and scalar invariance (see Section 3.3), these tests used item parcels and only two occasions per construct; future work with item-level data and additional waves should corroborate them (Boness et al., 2024). Relatedly, the six-month interval between waves may not have optimally captured the temporal dynamics of these relationships. The effects of parental phubbing on adolescent cognition and behavior may operate on different timescales: some effects may emerge within weeks, whereas others may require longer periods to manifest. Future work should consider varying the spacing between assessment waves to better capture these temporal processes (J. Liu et al., 2025).

## 6. Contributions and Practical Implications

This study extends prior cross-sectional evidence by examining how parental phubbing was prospectively associated with PMPU among Chinese adolescents one year later through core self-evaluation, and further elucidates the moderating role of peer relationships in this mediation process. First, the results of the longitudinal mediation analysis align with Davis’ cognitive-behavioral model, indicating that parental phubbing, as a distal factor, was not only directly associated with PMPU but was also indirectly associated with it through a proximal factor, namely maladaptive cognition embodied in core self-evaluation. This study provides empirical evidence for Davis’ cognitive-behavioral model specifically in the field of PMPU. Second, the results of the moderated mediation model test are consistent with ecological systems theory, which holds that both the family environment (i.e., parental phubbing) and the social environment (i.e., peer relationships) can be independently associated with adolescent development, and that furthermore, these two environments are interdependent and jointly associated with adolescents’ cognition (i.e., core self-evaluation) and behavior (i.e., PMPU). This study also offers guidance that may help mitigate the adverse outcomes associated with parental phubbing and adolescent PMPU, from the perspectives of core self-evaluation and peer relationships, with the aim of reducing the likelihood of PMPU among adolescents.

## 7. Conclusions

This study provides longitudinal evidence that parental phubbing was prospectively associated with subsequent PMPU in Chinese adolescents. Core self-evaluation significantly mediates this temporal link, and the residual direct effect is conditional on peer relationship quality. Furthermore, peer relationships were consistent with a protective-buffering role, attenuating the prospective links of parental phubbing to adolescents’ self-perception (i.e., core self-evaluation) and behavior (i.e., PMPU).

## Figures and Tables

**Table 1:** Attrition analyses comparing retained (*n* = 807) and attrited (*n* = 69) adolescents on Time 1 variables.

Variable	t	df	*p*	Cohen’s d
Age	0.62	874	0.536	0.09
SES	−0.81	874	0.418	0.11
T1 PP	1.04	874	0.299	0.14
T1 CSE	−0.77	874	0.442	0.10
T1 PMPU	0.55	874	0.583	0.08
T1 PR	−0.93	874	0.353	0.12
Gender	χ^2^(1) = 0.48		0.489	V = 0.023

**Table 2:** Descriptive Statistics and Correlations of the Main Variables.

Variables	1	2	3	4	5	6	7	8	9
Gender	1.00								
Age	−0.06	1.00							
SES	−0.04	0.01	1.00						
T1 PP	0.00	0.04	0.01	1.00					
T1 CSE	0.04	0.02	−0.01	−0.23 ***	1.00				
T2 CSE	0.01	−0.02	0.29 ***	−0.46 ***	0.40 ***	1.00			
T1 PMPU	−0.02	−0.03	0.02	0.30 ***	−0.09 *	−0.10 **	1.00		
T3 PMPU	0.01	−0.00	−0.14 ***	0.42 ***	−0.25 ***	−0.52 ***	0.19 ***	1.00	
T1 PR	0.02	−0.01	−0.00	−0.12 ***	0.22 ***	0.30 ***	−0.01	−0.25 ***	1.00
Mean	-	12.04	3.58	3.56	3.60	3.70	3.63	3.82	3.58
*SD*	-	1.44	1.35	0.95	0.93	0.81	0.87	0.90	1.02

Notes: Gender (1 = male, 0 = female), SES = socioeconomic status, PP = parental phubbing, CSE = core self-evaluation, PMPU = problematic mobile phone use, PR = peer relationships; T1 = Time 1, T2 = Time 2, T3 = Time 3; * *p* < 0.05, ** *p* < 0.01, *** *p* < 0.001.

**Table 3:** Longitudinal measurement invariance for core self-evaluation (T1–T2) and PMPU (T1–T3).

Construct	Model	χ^2^	df	CFI	RMSEA	SRMR	ΔCFI
CSE	Configural	46.2	19	0.972	0.043	0.039	—
CSE	Metric	52.8	22	0.968	0.042	0.045	0.004
CSE	Scalar	61.4	25	0.962	0.043	0.050	0.006
PMPU	Configural	55.1	19	0.963	0.051	0.044	—
PMPU	Metric	62.7	22	0.959	0.049	0.049	0.004
PMPU	Scalar	72.4	25	0.952	0.049	0.054	0.007

**Table 4:** Conditional indirect effects of T1 parental phubbing on T3 PMPU (via T2 core self-evaluation) and the index of moderated mediation (Bootstrap = 5000).

Level of Peer Relationships	Conditional Indirect Effect	Bootstrap 95% CI
Low PR (−1 SD)	0.20	[0.15, 0.24]
Mean PR	0.12	[0.10, 0.15]
High PR (+1 SD)	0.05	[0.03, 0.07]
Index of moderated mediation	−0.074	[−0.097, −0.054]

Note. Bootstrap = 5000 resamples. Continuous variables were standardized (M = 0, SD = 1) before estimation; the conditional indirect effects and the index of moderated mediation are on this standardized metric, with bias-corrected bootstrap confidence intervals on the same metric. Gender was retained on its original 0/1 scale.

**Table 5:** Exploratory sensitivity checks for school clustering (Mplus TYPE = COMPLEX; clustering by school; only three clusters).

Path	β (Cluster-Robust)	Robust 95% CI
X (T1 PP) → M (T2 CSE), mean PR	−0.38	[−0.47, −0.29]
M (T2 CSE) → Y (T3 PMPU)	−0.32	[−0.43, −0.21]
X (T1 PP) → Y (T3 PMPU), mean PR	0.23	[0.14, 0.32]
X × PR → M	0.23	[0.15, 0.31]
X × PR → Y	−0.14	[−0.22, −0.06]

Note. With only three schools, the sandwich correction cannot be treated as fully reliable; point estimates are reported to assess stability and do not demonstrate that clustering-related inferential concerns are resolved.

## Data Availability

The individual-level data presented in this study are not publicly available. They were collected from minors under a written parental informed-consent procedure that legally restricts the data to use within this research project, prohibits release to any third party, and requires encrypted storage with access limited to the research team; the individual-level dataset therefore cannot be shared. Aggregate results, the Mplus input syntax, and the annotated Mplus output are provided in the Supplementary Materials, and further aggregate information may be requested from the corresponding author subject to these restrictions.

## Supplementary Materials

The following supporting information can be downloaded at: https://www.mdpi.com/article/10.3390/bs16101947/s1, Syntax S1: Mplus input syntax for the simple mediation model; Syntax S2: Mplus input syntax for the moderated mediation model; Syntax S3: Mplus input syntax for the longitudinal measurement-invariance models; Syntax S4: Mplus input syntax for the fully latent robustness model; Syntax S5: Mplus input syntax for the cluster-robust (TYPE = COMPLEX) sensitivity model; Syntax S6: Mplus input syntax for the school fixed-effect model; Syntax S7: Mplus input syntax for the no-covariate model; Syntax S8: Mplus input syntax for the saturated cross-lagged sensitivity model; an annotated Mplus output summary is also provided.

## Appendix A. Item-to-Parcel Assignments

**Table A1:** Three-parcel assignments used in the fully latent robustness analysis (Section 3.6.1).

Scale (Number of Items)	Parcel 1	Parcel 2	Parcel 3
Parental phubbing (9)	1, 6, 7	2, 5, 8	3, 4, 9
Core self-evaluation (10)	1, 6, 7, 10	2, 5, 8	3, 4, 9
PMPU (17)	2, 5, 8, 11, 14, 17	3, 4, 9, 10, 15, 16	1, 6, 7, 12, 13
Peer relationships (18)	1, 6, 7, 12, 13, 18	2, 5, 8, 11, 14, 17	3, 4, 9, 10, 15, 16

**Table A2:** Four-parcel assignments used in the longitudinal measurement-invariance tests (Section 3.3).

Scale (Number of Items)	Parcel 1	Parcel 2	Parcel 3	Parcel 4
Core self-evaluation (10)	1, 8, 9	2, 7, 10	3, 6	4, 5
PMPU (17)	1, 8, 9, 16, 17	2, 7, 10, 15	3, 6, 11, 14	4, 5, 12, 13

Note: Within each scale, items were ordered by their item-total contribution and allocated to parcels in an alternating (serpentine), loadings-balanced sequence so that the parcels contain comparable numbers of items and comparable average factor loadings. Numbers denote the item numbers within each scale. The three-parcel allocation is reserved for the fully latent robustness analysis (matched product indicators), and the four-parcel allocation for the longitudinal invariance tests. As stated in Section 3.3 and Section 3.6.1, parcel-level invariance does not establish item-level measurement invariance or item-level construct validity.
